# Prox1 induces new lymphatic vessel formation and promotes nerve reconstruction in a mouse model of sciatic nerve crush injury

**DOI:** 10.1111/joa.13247

**Published:** 2020-06-09

**Authors:** Fan‐Wei Meng, Xue‐Ning Jing, Gui‐Hong Song, Lin‐Lin Jie, Fang‐Fang Shen

**Affiliations:** ^1^ Department of Anatomy and Physiology Shandong College of Traditional Chinese Medicine Yantai China

**Keywords:** lymphatic vessels formation, LYVE‐1, Prox1, sciatic nerve injury

## Abstract

The peripheral nervous system lacks lymphatic vessels and is protected by the blood–nerve barrier, which prevents lymphocytes and antibodies from entering the neural parenchyma. Peripheral nerve injury results in degeneration of the distal nerve and myelin degeneration causes macrophage aggregation, T lymphocyte infiltration, major histocompatibility complex class II antigen expression, and immunoglobulin G deposition in the nerve membrane, which together result in nerve edema and therefore affect nerve regeneration. In the present paper, we show myelin expression was absent from the sciatic nerve at 7 days after injury, and the expression levels of lymphatic vessel endothelial hyaluronan receptor 1 (LYVE‐1) and Prospero Homeobox 1 (Prox1) were significantly increased in the sciatic nerve at 7 days after injury. The lymphatic vessels were distributed around the myelin sheath and co‐localized with lymphatic endothelial cells. Prox1 induces the formation of new lymphatic vessels, which play important roles in the elimination of tissue edema as well as in morphological and functional restoration of the damaged nerve. This study provides evidence of the involvement of new lymphatic vessels in nerve repair after sciatic nerve injury.

## Graphical abstract

The peripheral nervous system lacks lymphatic vessels and is protected by the blood–nerve barrier. In the present study, increased expression of lymphatic vessel endothelial hyaluronan receptor 1 (LYVE‐1) and Prospero Homeobox 1 (Prox1) were observed in the sciatic nerve 7 days after injury, suggesting the presence of lymphatic vessels. Therefore, this study provides evidence of new lymphatic vessels involvement (mediated by Prox1) in nerve repair after sciatic nerve injury.

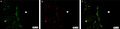

## 1 INTRODUCTION

The peripheral nervous system lacks lymphatic vessels and is protected by the blood–nerve barrier, which prevents lymphocytes and antibodies from entering the neural parenchyma (Schwartz *et al*. 1982). However, recent studies have found that peripheral nerve injury results in degeneration of the distal nerve (Ustun *et al*. 2017; Shefa and Jung, 2019). Myelin degeneration causes macrophage aggregation, T lymphocyte infiltration, major histocompatibility complex (MHC) class II antigen expression, and immunoglobulin G (IgG) deposition in the nerve membrane, which together result in nerve edema and therefore affect nerve regeneration (Willison *et al*. 2002). In addition, some studies have shown greater immunoglobulin deposits as well as poor nerve regeneration and functional recovery after severe nerve injury (Xiao *et al*. 2016; Yuan and Feng, 2016). These findings suggest that the local immune response after nerve injury has an inhibitory effect on nerve regeneration (Ansselin and Pollard, 1990; Navarro *et al*. 2007). On the other hand, peripheral nerve regeneration is dependent on both injured axons and non‐neuronal cells, such as Schwann cells, endoneurial fibroblasts and macrophages, which provide a supportive microenvironment for successful regrowth of the proximal nerve fiber ending (Caillaud *et al*. 2019).

The lymphatic system plays an important role in the immune system and fluid balance. Disruption of the lymphatic system leads to poor fluid drainage (especially in inflammatory tissues), which results in acute edema (Jamieson *et al*. 2005; Shin and Rockson, 2008; Swartz *et al*. 2008). The injury causes hypertonicity of the blood vessels and aggregation of neutrophils, macrophages, and other reactive cells that help in the clearing of dead cells and debris and promote angiogenesis (Nahrendorf *et al*. 2007; Barbay *et al*. 2015). However, several inflammatory factors and free radicals were also found to inhibit lymphatic function, resulting in lymphatic obstruction, edema, and chronic inflammation (Zawieja *et al*. 1991; Aldrich and Sevick‐Muraca, 2013). As lymphatic vessels are absent from the peripheral nerves and there have been no reports of lymphatic vessel involvement in nerve repair after damage, it was worth noting that edema was clinically apparent after peripheral nerve injury (Olmarker *et al*. 1989).

The formation of new lymphatic vessels is an important link between inflammation and the repair of damaged tissues (Watari *et al*. 2008). Prospero Homeobox 1 (Prox1) and vascular endothelial growth factor receptor 3 (VEGFR‐3) are known to play important roles in inflammation‐induced lymphangiogenesis [formation of new lymphatic vessels (Hong *et al*. 2002; Wigle *et al*. 2002)], and the nuclear factor‐κB (NF‐κB) family of transcription factors are key factors regulating inflammation‐induced transcription (Karin, 2006). Recent studies reported that inflammation induces increased expression of VEGFR‐3 on the lymphatic endothelium, and this effect is mediated by NF‐κB and Prox1. This results in a significant increase in the binding affinity of VEGFR‐3 to vascular endothelial growth factor (VEGF)‐D and VEGF‐D on lymphatic endothelial cells, leading to lymphangiogenesis (Olmarker *et al*. 1989).

Evidence has also shown that adequate intraneural vascularization is found concurrently with the regeneration of nerves (Mizisin and Weerasuriya, 2011; Goedee *et al*. 2013). Therefore, the present study investigated the expression of Prox1 after sciatic nerve crush injury as well as the presence of new lymphatic vessels in the sciatic nerve and their effects on nerve regeneration and functional recovery.

## 2 METHODS

### 2.1 Experimental animals

Specific pathogen‐free male Kunming mice, weighing 18–26 g were provided by Shandong University Laboratory Animal Centre, license number: SCXK (Lu) 2010‐0001 and laboratory animal license number: SYXK (Lu) 2010‐0011. The mice were housed at 21–23°C on a 12‐h light/dark cycle with *ad libitum* access to food and water. All experimental protocols were approved by the Shandong College of Traditional Chinese Medicine Ethics Committee. All procedures performed in studies involving animals were in accordance with the ethical standards of the institution or practice at which the studies were conducted.

### 2.2 Mouse model of sciatic nerve crush injury

Forty mice were randomly divided into control groups (days 7 and 14) and two sciatic nerve injury groups analyzed after 7 and 14 days (*n* = 10/group). The mice were deeply anesthetized for surgery using a combination of ketamine (50 mg/kg) and xylazine (40 mg/kg). The mice were placed in prone position on mouse plates, and all limbs were taped down. Along the midline of the back of the thigh, an incision was made in the skin. The biceps femoris and semimembranosus muscle were separated, revealing the sciatic nerve at the edge of the gluteus maximus. A length of 1 cm of the sciatic nerve was clamped and unclamped using a vascular clamp for a duration of 10 s each time, for a total of three times. Non‐invasive clamps (using #0 surgical sutures) were used to mark the upper and lower end of the sciatic nerve. After the surgery, the skin incision was sutured using #7 surgical sutures. For the control group, the sciatic nerve was exposed without clamping.

### 2.3 Effects of sciatic nerve injury on the myelin sheath

The four groups of mice were killed at their respective time points (7 or 14 days after surgery), and the control mice were killed on days 7 and 14 after surgery. The clamped sciatic nerve of the right thigh was removed, and the tissues were fixed in 4% formaldehyde and embedded in paraffin. The paraffin blocks were then sectioned at 4 µm, stained with Luxol fast blue (myelin stain), and viewed under a DP‐72 optical microscope (Olympus). Some tissues were also fixed in 4% paraformaldehyde solution for 3 h before immersion in 30% sucrose (in phosphate‐buffered saline) for dehydration. The tissues were rapidly frozen in liquid nitrogen and sectioned at 20 µm. The sections were further fixed in 1% osmium tetroxide for 30 min, dehydrated using ethanol, and embedded in EPON 812. The sections were then cut into ultra‐thin slices and viewed under an HC‐1 electron microscope (Hitachi).

### 2.4 Protein expression of lymphatic vessel endothelial hyaluronan receptor 1, Prox1 and myelin basic protein in the sciatic nerve after injury

The paraffin‐embedded sections of mouse tissues were deparaffinized and subjected to antigen retrieval in 0.01 M citrate buffer at 121°C for 15 min. Endogenous peroxidase was inactivated using 3% of hydrogen peroxide. The sections were incubated in rabbit anti‐mouse Prox1 antibody (diluted 1:100; Boster Biological Technology) or goat anti‐mouse lymphatic vessel endothelial hyaluronan receptor 1 (LYVE‐1) antibody (diluted 1:100; R&D Systems) at 4°C for 24 h, followed by incubation in horseradish peroxidase‐labeled goat anti‐rabbit IgG or rabbit anti‐goat IgG. 3’3’‐Diaminobenzidine (DAB) staining was performed using a DAB immunostaining kit (Boster Biological Technology), and the sections were viewed under a DP‐72 optical microscope (Olympus). The sections were viewed at 400 × magnification, and the images for each tissue section were captured at 50 different points. Semi‐quantitative analysis was performed using Image‐Pro Plus 6.0, and the average optical density value was analyzed.

For double immunofluorescence staining, the paraffin‐embedded sections were deparaffinized and subjected to antigen retrieval as described above. The sections were incubated in rabbit anti‐mouse Prox1 antibody (diluted 1:100; Boster Biological Technology) and goat anti‐mouse LYVE‐1 antibody (diluted 1:100; R&D Systems), or rabbit anti‐mouse myelin basic protein (MBP; diluted 1:100; Boster Biological Technology) and goat anti‐mouse LYVE‐1 antibody (diluted 1:100; R&D Systems) at 4°C for 24 h. Next, the sections were incubated in fluorescein isothiocyanate (FITC) donkey anti‐goat IgG (Boster Biological Technology) and Cy3‐labeled donkey anti‐rabbit IgG (Boster Biological Technology). The tissue sections were viewed under DP‐72 fluorescence microscope (Olympus) and images captured.

### 2.5 Statistical analysis

All statistical analysis was performed using SPSS 19.0 statistical software. Comparison between groups was performed using analysis of variance with Tukey *post hoc* analysis. A value of *P* < 0.05 was considered statistically significant.

## 3 RESULTS

### 3.1 Myelin reduction and morphological changes on day 7 after sciatic nerve injury

**Figure 1:**
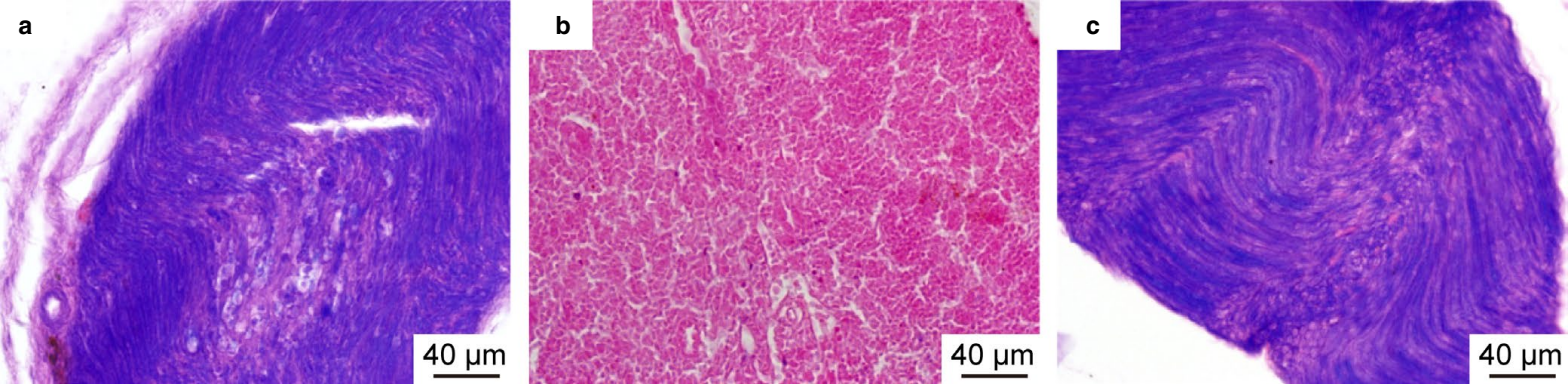
Luxol blue staining of the sciatic nerve: (A) control, (B) 7 days after sciatic nerve injury, and (C) 14 days after sciatic nerve injury. The nerve fiber and myelin sheath were clearly seen in the sciatic nerve of the controls. However, only the nerve fiber was observed on the 7th day after nerve injury, with absence of the myelin sheath in the sciatic nerve. On the 14th after nerve injury, the myelin sheath had reappeared. Blue, myelin; red, nerve fiber.

In the control group, the nerve fiber (red) and myelin sheath (blue) were clearly seen in the sciatic nerve (Figure 1a). However, on the 7th day after nerve injury, the nerve fiber (red) was observed, with degeneration of the myelin sheath in the sciatic nerve (Figure 1b). On the 14th after nerve injury, the myelin sheath (blue) had reappeared (Figure 1c), and the distance between the nerve membranes had returned to normal (data not shown). Note that as the sciatic nerve tissues were very small, paraffin embedding was difficult, which resulted in variable orientation and positioning of the tissues in the paraffin wax. Thus, obtaining a consistent set of sections for each group was difficult, and this accounts for the different orientations in Figure 1a–c.

**Figure 2:**
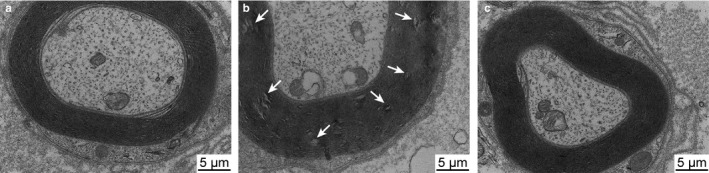
Transmission electron microscopy images of the sciatic nerve fibers: (A) control, (B) 7 days after sciatic nerve injury, and (C) 14 days after sciatic nerve injury. The control group showed a tightly packed and clearly defined myelin sheath in the sciatic nerve. However, on the 7th day after nerve injury, the myelin sheath showed morphological changes, appearing disorganized and folded. On the 14th after the nerve injury, most of the myelin structure had returned to normal. White arrows indicate morphological changes in the myelin

Electron microscopy images showed a tightly packed and clearly defined myelin sheath in the sciatic nerve of the control groups (Figure 2a). However, on the 7th day after nerve injury, the myelin sheath showed morphological changes, appearing disorganized and folded (Figure 2b). On the 14th after the nerve injury, most of the myelin structure had returned to normal (Figure 2c).

### 3.2 Increased protein expression of LYVE‐1 on day 7 after sciatic nerve injury

**Figure 3:**
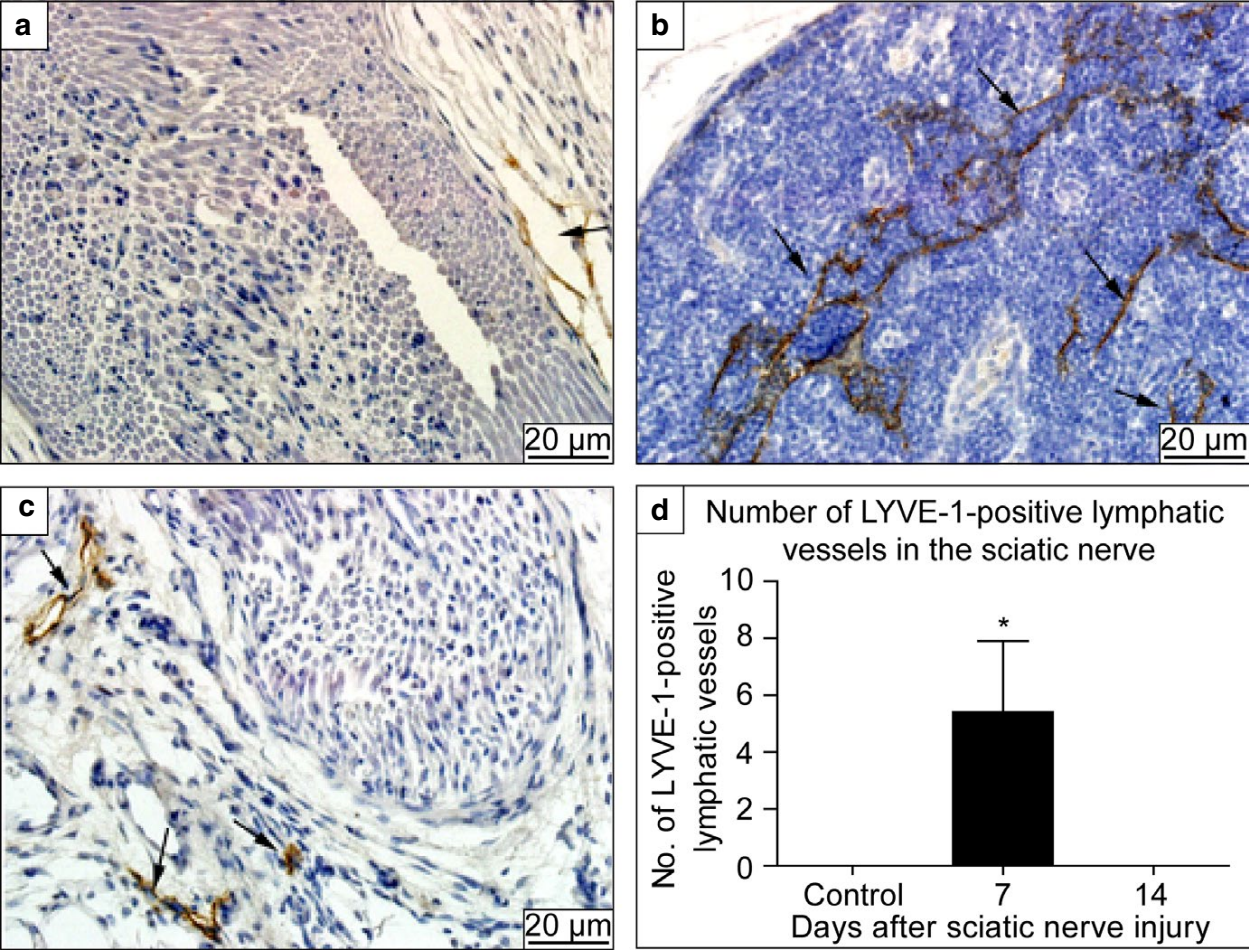
Expression of lymphatic vessel endothelial hyaluronan receptor 1 (LYVE‐1) in the sciatic nerve: (A) control, (B) 7 days after sciatic nerve injury, and (C) 14 days after sciatic nerve injury. (D) Number of LYVE‐1‐positive cells (lymphatic vessels). Black arrows: LYVE‐1‐positive lymphatic vessels. The lymphatic vessels are located outside the sciatic nerve in the images in (A) and (C), whereas, the lymphatic vessels are located in the sciatic nerve in the image in (B). The number of lymphatic vessels was increased significantly in the sciatic nerve at 7 days after injury, compared to that observed in the control group. No significant difference was observed between the control group and the injury model at 14 days after injury. *P < 0.05. Asterisk indicates areas within the nerve membranes

In the control group, tubular LYVE‐1‐positive cells were observed in the outer nerve membrane. No LYVE‐1‐positive cells were observed within the nerve membranes (Figure 3a). However, on the 7th day after nerve injury, a large number of tubular LYVE‐1‐positive cells were observed within the membrane of the sciatic nerve (Figure 3b), and the staining pattern was similar to that for the lymphatic vessels surrounding the epineurium. On the 14th day after nerve injury, no LYVE‐1‐positive cells were observed within the membrane of the sciatic nerve, and the lymphatic vessels were distributed outside the epineurium (Figure 3c). Statistical analysis showed that the expression of LYVE‐1‐positive cells in the sciatic nerve on the 7th day after injury was significantly higher, compared to that in the control group and at 14 days after injury (Figure 3d). No significant difference was observed between the control and the injury model at 14 days after injury.

### 3.3 Increased protein expression of Prox1 on day 7 after sciatic nerve injury

**Figure 4:**
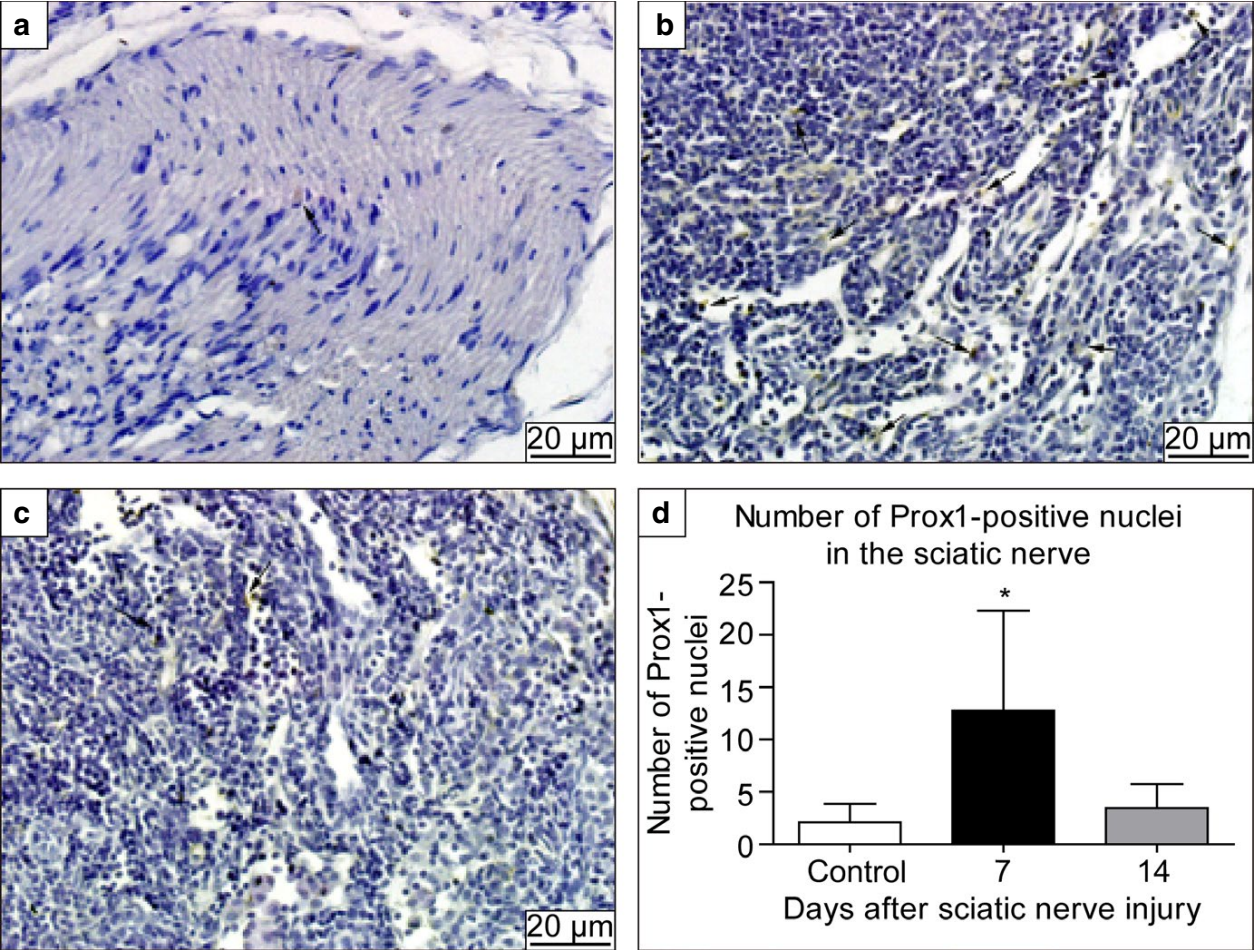
Expression of Prospero Homeobox 1 (Prox1) in the sciatic nerve. (A) Control, (B) 7 days after sciatic nerve injury, and (C) 14 days after sciatic nerve injury. (D) Number of Prox1‐positive cells. Black arrows: Prox1‐positive nuclei. The Prox1‐positive nuclei were mostly observed in the tubular endothelial cells. The number of Prox1‐positive nuclei were significantly greater in the sciatic nerve at 7 days after injury, compared to that in the control group. No significant difference was observed between the control group and injury model at 14 days after injury. *P < 0.05.

A few Prox1‐positive cells were observed in the sciatic nerve of the control group (Figure 4a). However, on the 7th day after nerve injury, a large number of Prox1‐positive nuclei were seen within the membrane of the sciatic nerve, with some distribution observed in the endothelial cells in certain parts of the lymphatic vessels (Figure 4b). On the 14th day after nerve injury, there were a decreased number of Prox1‐positive cells (Figure 4c). Statistical analysis showed that the expression of Prox1‐positive nuclei in the sciatic nerve was significantly increased on the 7th day after injury, compared to that in the control group and at 14 days after injury (Figure 4d). No significant difference was observed between the control and the injury model at 14 days after injury.

### 3.4 Expression of LYVE‐1‐positive lymphatic vessels and MBP‐positive myelin sheath at 7 and 14 days after nerve injury, and at 7 and 14 days after control surgery

**Figure 5:**
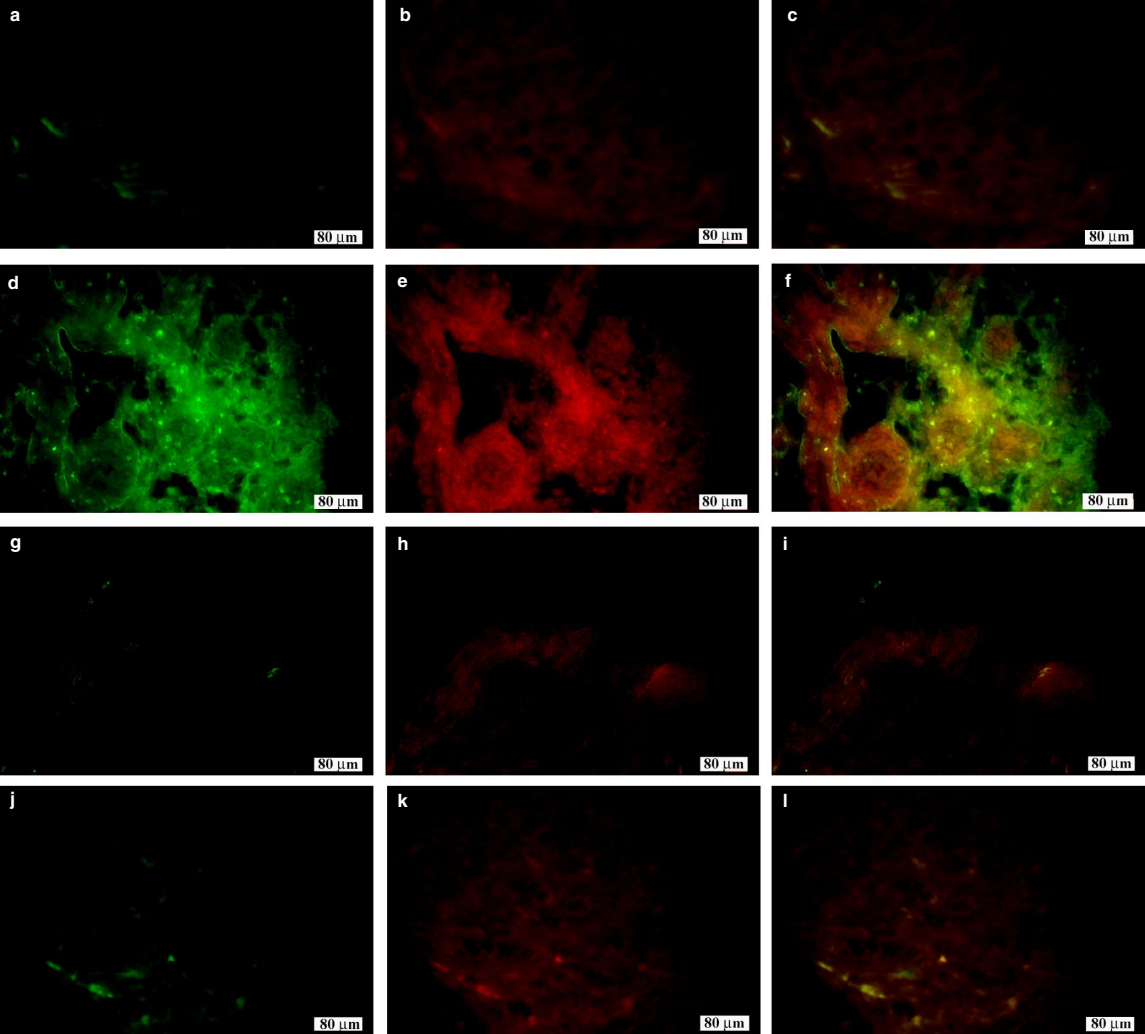
Expression of lymphatic vessel endothelial hyaluronan receptor 1 (LYVE‐1)‐positive lymphatic vessels and myelin basic protein (MBP)‐positive myelin sheath in the sciatic nerve at 7 and 14 days after control surgery, and at 7 and 14 days after injury. (A–C) 7 days in control group, (D–F) 7 days after injury, (G–I) 14 days in control group and (J–L) 14 days after injury. At 7 days after nerve injury, lymphatic vessels (green) were distributed around the myelin sheath (red), while at 7 and 14 days in the control group, and at 14 days after nerve injury, the lymphatic vessels were seen distributed outside the nerve, with no distribution within the myelin sheath. Green, LYVE‐1; red, MBP.

The relationship between LYVE‐1‐positive lymphatic vessels and myelin sheath was examined using double immunolabeling with a myelin marker, MBP. The LYVE‐1‐positive cells were labeled in green and the MBP‐positive cells were labeled in red. At 7 days after the control surgery, the lymphatic vessels were distributed outside the nerve (Figure 5a–c). However, at 7 days after nerve injury, distribution of lymphatic vessels was observed around the myelin sheath (Figure 5d–f). At 14 days after control surgery or nerve injury, the result was similar to that after 7 days in the control group, which showed distribution of lymphatic vessels outside the nerve, with no distribution within the myelin sheath (Figure 5g–i and Figure 5j–l, respectively).

### 3.5 Co‐localization of LYVE‐1‐positive lymphatic vessels with Prox1‐positive lymphatic endothelial cells on day 7 after sciatic nerve injury

**Figure 6:**
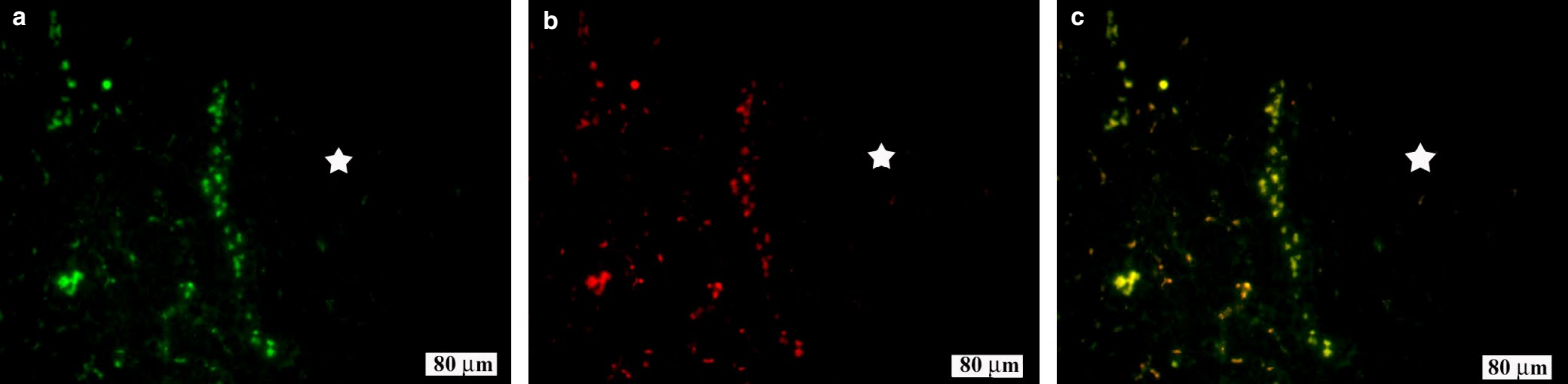
Co‐localization of lymphatic vessel endothelial hyaluronan receptor 1 (LYVE‐1)‐positive lymphatic vessels with Prospero Homeobox 1 (Prox1)‐positive endothelial nuclei in the sciatic nerve at 7 days after injury. (A) LYVE‐1 (green), (B) Prox1 (red), and (C) LYVE‐1 + Prox1+ (merged image), showing co‐localization of lymphatic vessels with endothelial cells (yellow). White star indicates areas within the nerve membranes.

The relationship between LYVE‐1‐positive lymphatic vessels and lymphatic endothelial cells was examined using double immunolabeling with a lymphatic endothelial cell marker, Prox1. The LYVE‐1‐positive cells were labeled in green (Figure 6a), and the Prox1‐positive cells were labeled in red (Figure 6b). The results showed that the lymphatic vessels were co‐localized with the lymphatic endothelial cells (Figure 6c).

## 4 DISCUSSION

Compression of the peripheral nerve causes damage to the nerve structure and function, which leads to sensory and motor dysfunction, such as carpal tunnel syndrome and cervical and lumbar disc herniation (Stoll *et al*. 2002). Clinically, treatment for peripheral nerve compression has achieved favorable outcomes; however, the pathological changes as well as the repair and regeneration mechanisms after peripheral nerve compression remain incompletely understood. In addition, protecting the pinched nerve, reducing secondary damage to the nerve, and restoring its conductive function are difficult problems that have yet to be completely solved in the clinical field. In the early stage of nerve compression, nerve ischemia, hypoxia, and microcirculatory disorders lead to the destruction of the blood–nerve barrier, causing nerve edema (Kaiser and Haninec, 2012). Research has indicated that the degree of neuroinflammatory responses after nerve injury is inversely correlated with functional recovery. Prolonged nerve compression results in nutrient deficiency of the nerve. This leads to thickening of the outer membrane, hyperplasia of the connective tissue, Wallerian degeneration, fibroblast invasion, adhesion formation and permanent scarring, and severe neurological impairment, which affects nerve conduction function (Rydevik *et al*. 1980; Bouldin *et al*. 1991; Aziz *et al*. 1999).

During the early stage of injury, macrophages proliferate and accumulate at the injury site, clearing the ruptured axons and myelin sheath via phagocytosis. At the same time, Schwann cells secrete various neurotrophic factors such as brain‐derived neurotrophic factor, nerve growth factor, fibroblast growth factor and leukemia inhibitory factor, forming bands of Bünger in the peripheral nerve. The neuronal cell body undergoes chromatolysis (axonal reaction) and develops growth cones on the surface of the retraction ball, which grow toward the target organs at a rate of 2–4 mm/day. The Schwann cells gradually wrap around the axons to form the regenerated myelin sheath (Funakoshi *et al*. 1993). Elimination of the inflammatory response and edema of the nerve in the early stage of injury could prevent myelin degeneration and fibrous scar formation in the later stage of the injury, which would be beneficial to the functional recovery of the nerve. However, how nerve edema is relieved and whether lymphatic vessels are involved in relieving nerve edema after injury have not been well studied. This is because it is generally believed that the peripheral nerves lack lymphatic vessels and are restricted to outside the neuroepithelium.

The lymphatic system plays several physiological and pathological roles, including regulation of interstitial fluid pressure, lipid absorption, immune surveillance, and reducing of inflammation (Tammela and Alitalo, 2010). The formation of new lymphatic vessels is dynamic during embryogenesis; however, it is relatively rare and selectively regulated in adulthood. Insufficient postpartum lymphatic development impairs immune function, leading to tissue edema (Adams and Alitalo, 2007). The formation of new lymphatic vessels is regulated by the homeobox transcription factor Prox1. Research has shown that Prox1 promotes lymphangiogenesis in adult inflammatory conditions (Wigle *et al*. 2002), and Prox1 siRNA injection in rats was found to inhibit lymphangiogenesis (Rho *et al*. 2015). During the early stages of embryonic development, VEGFR‐3 is expressed on vascular endothelial cells, and its ligand, VEGF‐C, plays an important role in the eventual establishment of lymphatic endothelial cells (Joukov *et al*. 1996; Wigle *et al*. 2002). VEGFR‐3 is a tyrosine kinase receptor mainly expressed on lymphatic endothelial cells. It is one of the key proteins in regulating lymphangiogenesis, and its ligands include VEGF‐C and VEGF‐D (Kukk *et al*. 1996). At the site of inflammation, various cell types such as macrophages, dendritic cells, neutrophils, mast cells, and fibroblasts, secrete large amounts of VEGF‐C and VEGF‐D, which enhances the binding of VEGF‐C and VEGF‐D to VEGFR‐3 (Ristimaki *et al*. 1998; Cursiefen *et al*. 2004; Baluk *et al*. 2005). Although a large body of evidence supports the notion of inflammation‐induced lymphangiogenesis, the molecular mechanisms of this association have not been elucidated in detail. In addition, an *in vivo* study showed that NF‐κB and Prox1 activate transcription of VEGFR‐3, suggesting important roles for NF‐κB and Prox1 in the regulation of VEGFR‐3‐dependent lymphangiogenesis (Flister *et al*. 2010).

In the present study, morphological changes and reduction of myelin were observed 7 days after sciatic nerve injury, together with the distribution of lymphatic vessels around the myelin. Increased expression of Prox1 was also observed in the sciatic nerve at 7 days after injury. Prox1 is known to play a key regulatory role in lymphatic vessel‐mediated elimination of tissue edema and morphological and functional restoration of damaged nerve. In addition, Prox1 induced lymphatic endothelial cell proliferation around the sciatic nerve, resulting in formation of new lymphatic vessels, which was observed through an increase in LYVE‐1 expression and co‐localization of Prox1 and LYVE‐1 at 7 days after sciatic nerve injury. This is consistent with a study that showed an increase in the number of vessels after sciatic nerve crush injury (Caillaud *et al*. 2019). The new lymphatic vessels could effectively reduce tissue edema by increasing the removal of excess interstitial fluid, inflammatory cells, and myelin debris, and therefore, promote the formation of new myelin by the glial cells to enhance the functional recovery of the sciatic nerve. This is supported by the results observed on day 14 after nerve injury, when the myelin sheath had reappeared and the structure had returned to normal.

In conclusion, our results showed increased expression of Prox1 and the presence of lymphatic vessels (LYVE‐1) at 7 days after sciatic nerve injury. This increase in lymphatic vessels helped to eliminate edema and restore the morphology and function of the damaged nerve. This study provides evidence of the presence of new lymphatic vessels in nerve repair after sciatic nerve injury in mice. Further research using Prox1 siRNA is necessary to elucidate the physiological role of Prox1 in the sciatic nerve after injury.

## CONFLICTS OF INTEREST

None declared.

## AUTHOR CONTRIBUTION

F.W.M. conceived and designed research. X.N.J., L.L.J. and F.F.S. collected data and conducted research. F.W.M. and G.H.S. analyzed and interpreted data. F.W.M. wrote the initial paper; All authors revised the paper, read and approved the final manuscript.

## Data Availability

The datasets generated and analyzed during the present study are available from the corresponding author upon reasonable request.
